# Supplementary material for: Reproducible and sensitive micro-tissue RNA sequencing from formalin-fixed paraffin-embedded tissues for spatial gene expression analysis
Source: Sci Rep. 2022 Nov 14;12:19511. doi: 10.1038/s41598-022-23651-6 (PMC9663554; doi:10.1038/s41598-022-23651-6)
Supplement: Supplementary file 1 — Supplementary Figures. [file 41598_2022_23651_MOESM1_ESM.pdf]

Supplementary Information

Reproducible and sensitive micro-tissue RNA sequencing from formalin-fixed paraffin-embedded tissues for spatial gene expression analysis

Hiroko Matsunaga[1], Koji Arikawa[1], Miki Yamazaki[2,3], Ryota Wagatsuma[2,3], Keigo Ide[2,3], Ashok Zachariah Samuel [1], Kazuya Takamochi[4], Kenji Suzuki[4], Takuo Hayashi[5], Masahito Hosokawa[1,2,3,6], Hideki Kambara[1,7], and Haruko Takeyama[1,2,3,6*]

[1] Research organization for Nano and Life Innovation, Waseda University

[2] Department of Life Science and Medical Bioscience, Waseda University

[3] Computational Bio Big-Data Open Innovation Laboratory, AIST-Waseda University

[4] Department of Thoracic Surgery, Juntendo University School of Medicine

[5] Department of Human Pathology, Graduate School of Medicine, Juntendo University

[6] Institute for Advanced Research of Biosystem Dynamics, Waseda Research Institute for Science and Engineering, Waseda University

[7]Frontier biosystems, Inc.

Corresponding author: Haruko Takeyama (haruko-takeyama@waseda.jp)

Supplementary Figure 1

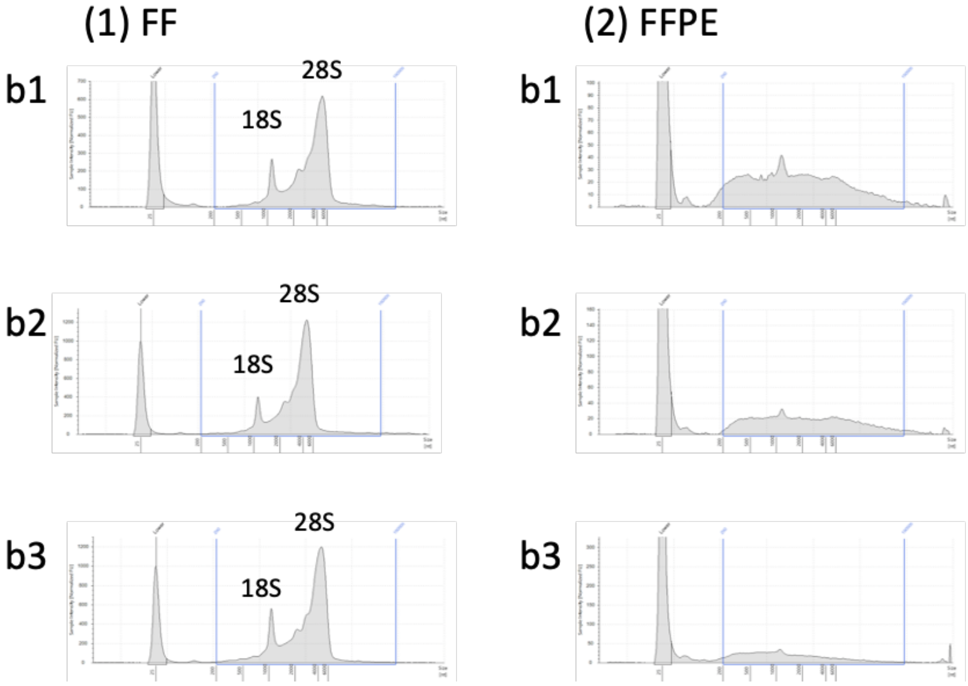

Supplementary Figure 1. Electropherogram of total RNA

The electrophoretic patterns of purified total RNA were obtained using a commercially available kit (RNeasy Mini Kit (QIAGEN, Hilden, Germany) or RNeasy FFPE Kit (QIAGEN)). Peaks of ribosomal RNA (18S and 28S) could be detected in total RNA extracted from FF tissues, but not in that from FFPE tissues. Compared with that in FF tissues, total RNA from FFPE tissue was spread uniformly over a wide range of base lengths, from long to short, indicating that the latter was degraded.

FF, fresh-frozen; FFPE, formalin-fixed paraffin-embedded.

Supplementary Figure 2

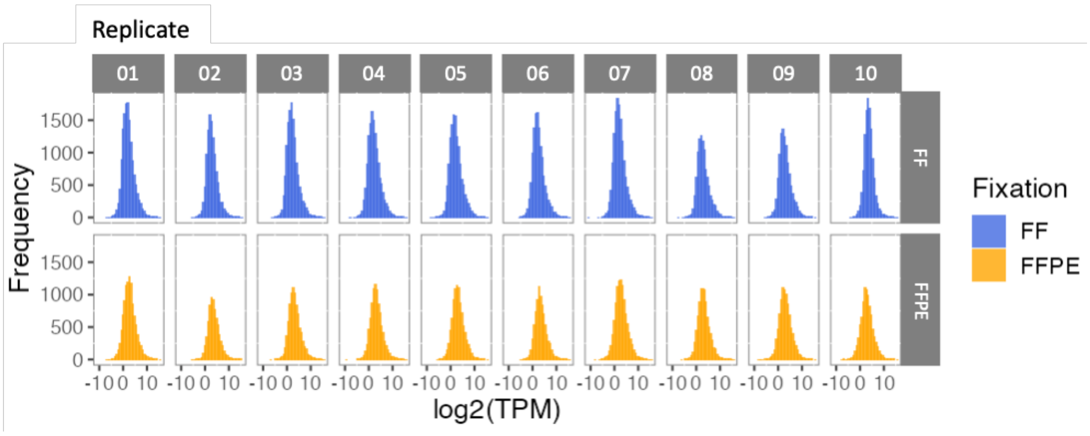

Supplementary Figure 2. Correlation of gene expression patterns

The frequency patterns of gene expression in the PuTi-spots (N = 10) obtained from FF and FFPE tissues were highly similar in form, except that the frequency was higher in FF tissues than in FFPE tissues. The frequency of expression is shown on the vertical axis, and the expression (log2(TPM)) on the horizontal axis.

PuTi, micro-punched tissue spot; FF, fresh-frozen; FFPE, formalin-fixed paraffin-embedded.

Supplementary Figure 3_1

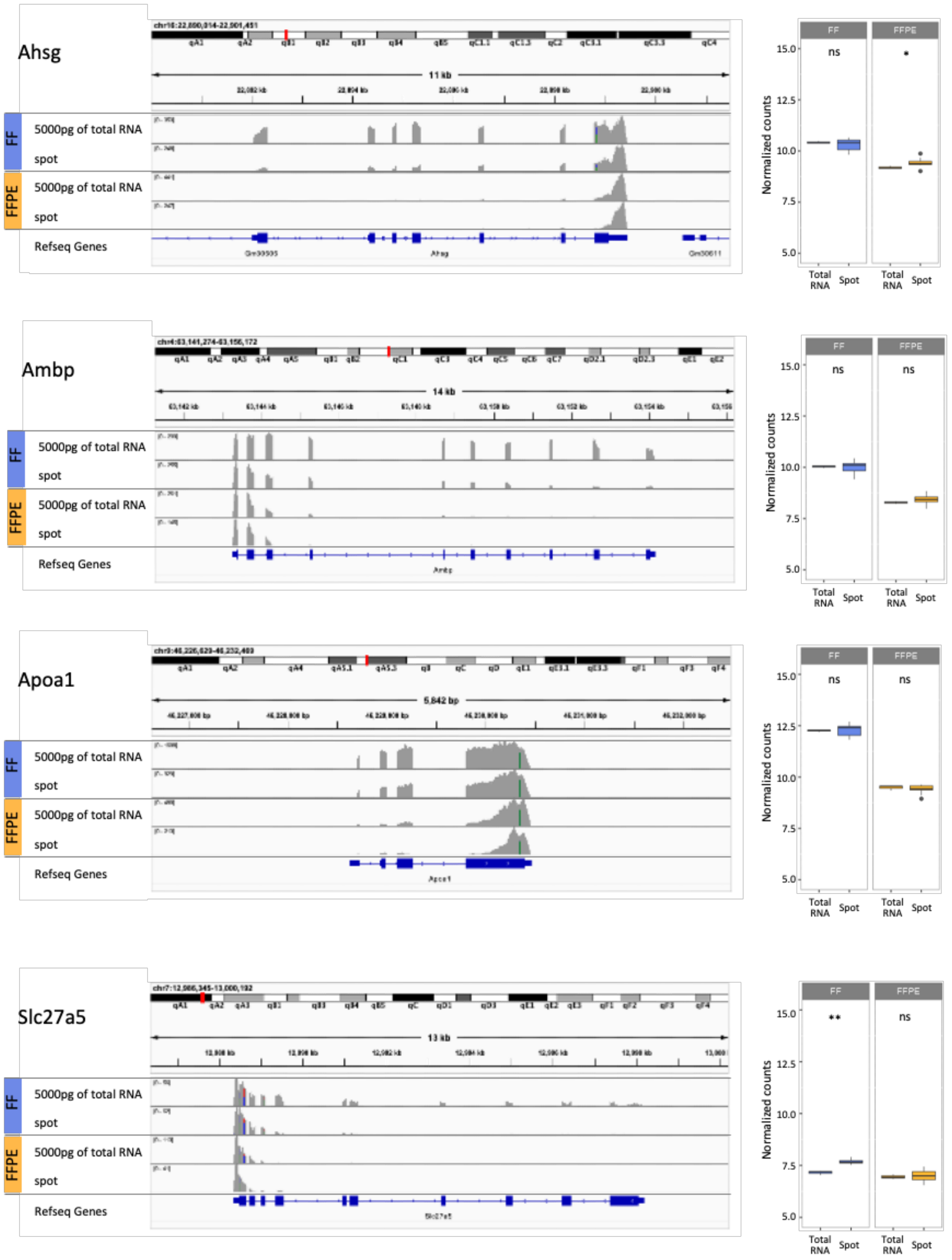

Supplementary Figure 3_2

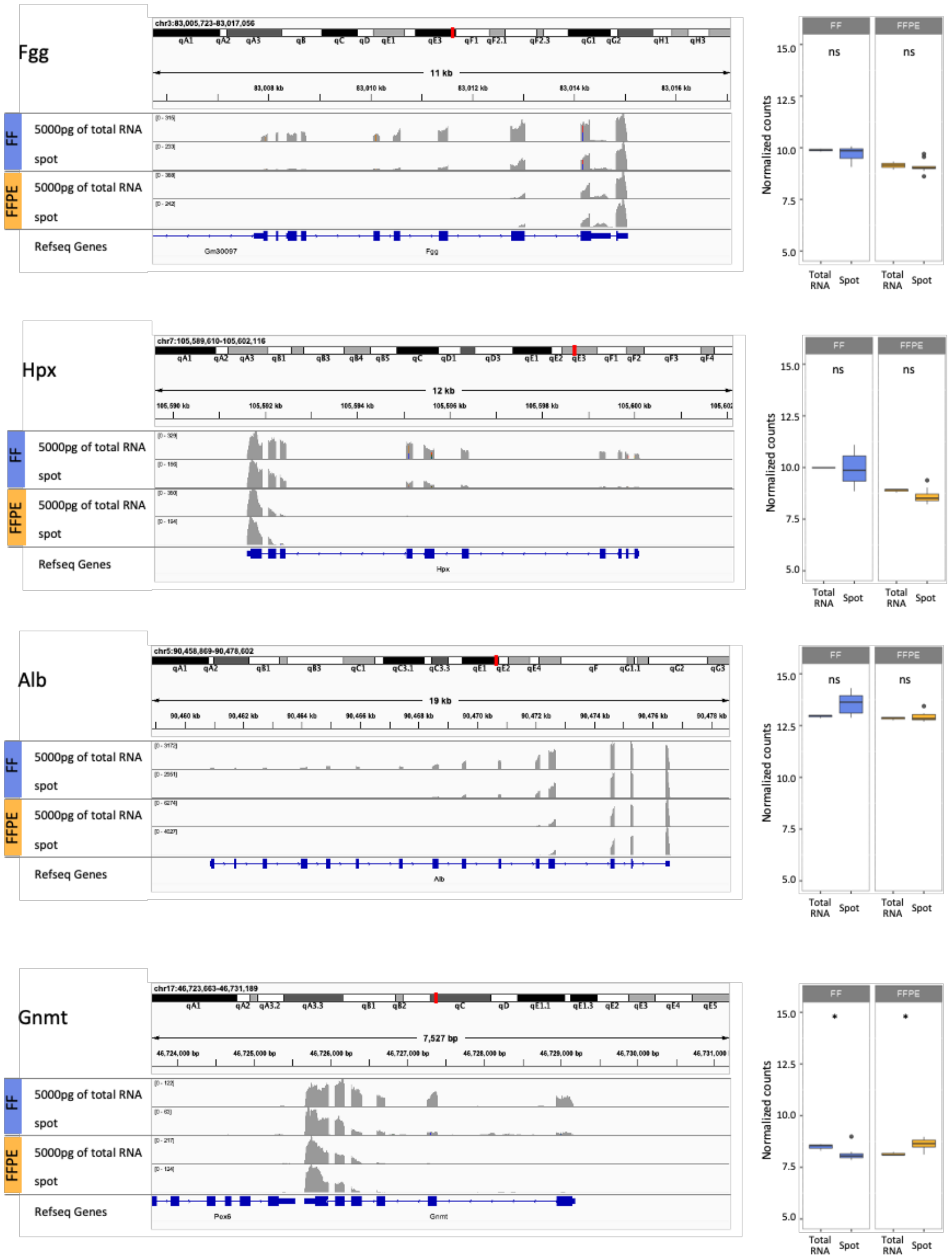

Supplementary Figure 3_3

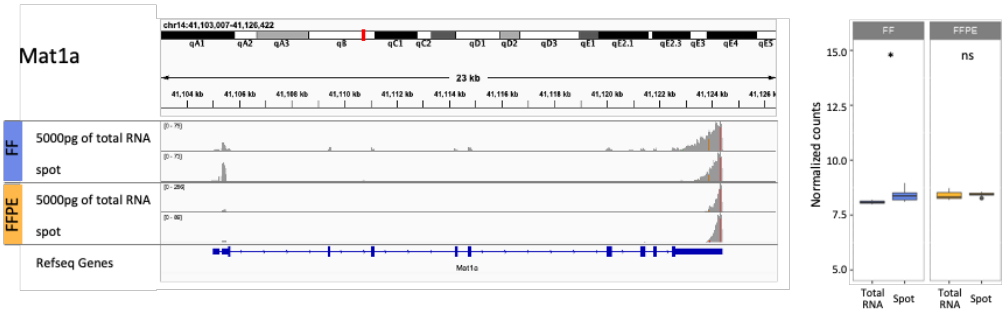

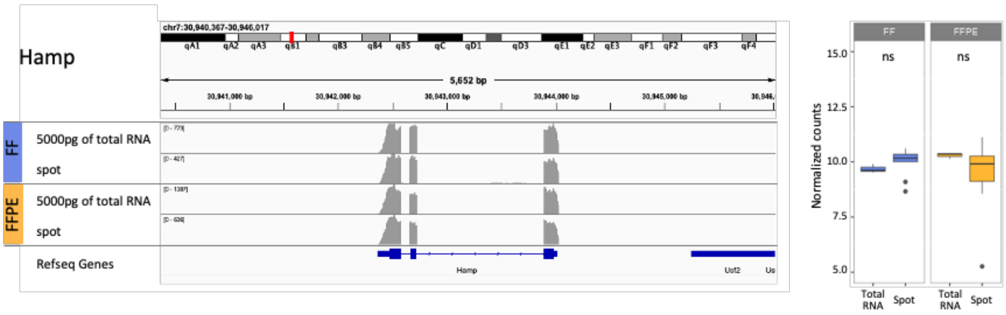

Supplementary Figure 3. Gene expression comparison between bulk tissue and the tissue spots

Alignment of reads to ten liver-specific genes (left), and comparison of normalized read counts between purified total RNA and PuTi-spots derived from FF or FFPE tissue (right boxplot). Read coverage was visualized using Integrative Genomics Viewer. The bottom of the column shows visualized exons (bold line and boxes) and introns (line). Sequencing reads derived from purified total RNA are more well-characterized for alignment, depending on the tissue preservation method. A greater number of reads were aligned at the 3'-end of each gene, and fewer reads were mapped toward the 5'-end. In both FF-derived samples (purified total RNA and PuTi-spots), the aligned region of reads was longer and extended toward the 5'-terminus. The difference in normalized read counts between FF and FFPE tissues could be attributed to the length of the alignment from the 3'-end.

PuTi, micro-punched tissue spot; FF, fresh-frozen; FFPE, formalin-fixed paraffin-embedded.

Supplementary Figure 4

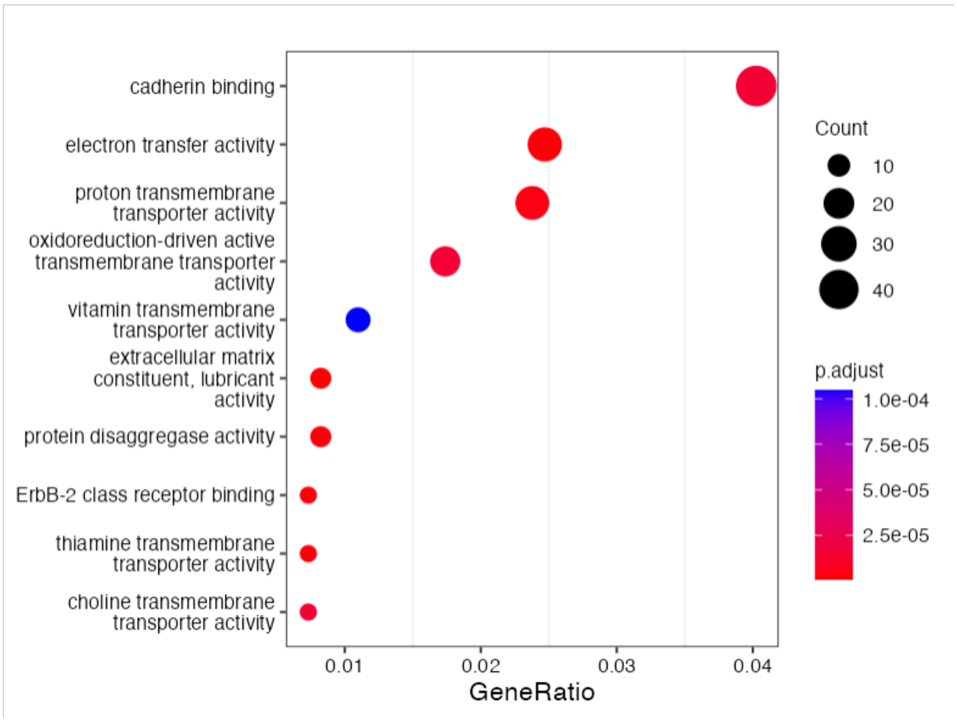

Supplementary Figure 4. Gene ontology enrichment analysis of upregulated genes in PuTi-spots collected from the tumor area. Molecular function-linked cadherin binding was enriched in tumor PuTi-spots (*p-adjust* = 1.51×10^{-5}), and the *S100P* and *TAGLN2* genes were included (Supplementary Table 1).

PuTi-spot, micro-punched tissue spot.
